# Even- and odd-chain saturated fatty acids in serum phospholipids are differentially associated with adipokines

**DOI:** 10.1371/journal.pone.0178192

**Published:** 2017-05-26

**Authors:** Kayo Kurotani, Masao Sato, Kazuki Yasuda, Kentaro Kashima, Shoji Tanaka, Takuya Hayashi, Bungo Shirouchi, Shamima Akter, Ikuko Kashino, Hitomi Hayabuchi, Tetsuya Mizoue

**Affiliations:** 1 Department of Epidemiology and Prevention, Center for Clinical Sciences, National Center for Global Health and Medicine, Tokyo, Japan; 2 Laboratory of Nutrition Chemistry, Department of Bioscience and Biotechnology, Faculty of Agriculture, Graduate School, Kyushu University, Fukuoka, Japan; 3 Department of Metabolic Disorder, Diabetes Research Center, National Center for Global Health and Medicine, Tokyo, Japan; 4 Graduate School of Nutrition and Health Science, Fukuoka Women’s University, Fukuoka, Japan; Medical University Innsbruck, AUSTRIA

## Abstract

**Background:**

Saturated fatty acids are generally thought to have detrimental effects on health. However, a recent study showed that even- and odd-chain saturated fatty acids had opposite associations with type 2 diabetes. Limited studies of Western populations examined the associations of circulating saturated fatty acids with adipokines, an important role in glucose metabolism.

**Objective:**

We examined the associations of saturated fatty acids in serum phospholipids with circulating levels of adipokines among a Japanese population.

**Design:**

A cross-sectional study was conducted among 484 Japanese employees (284 men and 200 women) aged 20–65 years. The serum fatty acid composition in the phospholipid fraction was measured by gas-chromatography. Serum leptin, adiponectin, plasminogen activator inhibitor-1 (PAI-1), resistin, and visfatin were measured using a Luminex suspension bead-based multiplexed array. Multiple linear regression analysis was performed to assess the association between saturated fatty acids and adipokines, with adjustment for potential confounding variables.

**Results:**

Even- and odd-chain saturated fatty acids were differentially associated with adipokines. Higher levels of even-chain saturated fatty acids (14:0 myristic, 16:0 palmitic, and 18:0 stearic acids) were associated with higher levels of resistin (P for trend = 0.048) and lower levels of adiponectin (P for trend = 0.003). By contrast, odd-chain saturated fatty acids (15:0 pentadecanoic and 17:0 heptadecanoic acids) showed inverse associations with leptin and PAI-1 (P for trend = 0.048 and 0.02, respectively). Visfatin was positively associated with both even- and odd-chain saturated fatty acids.

**Conclusions:**

The results suggest that even- and odd-chain saturated fatty acids are differentially associated with adipokine profile.

## Introduction

Contrary to the common belief that a reduction in dietary saturated fatty acids improves cardiovascular health, a recent meta-analysis of prospective cohort studies concluded that both a higher intake and circulating forms of saturated fatty acids are not harmful in terms of risk of coronary disease [1]. In the European Prospective Investigation into Cancer and Nutrition Study (EPIC) and the Norfolk Prospective Study, even-chain saturated fatty acid concentrations were associated with an increased risk of coronary heart disease, whereas odd-chain saturated fatty acid concentrations were associated with a decreased risk [2]. Similarly, the EPIC-InterAct showed that even-chain saturated fatty acids were positively associated with type 2 diabetes, whereas odd-chain saturated fatty acids were inversely associated with type 2 diabetes [3]. Thus, individual saturated fatty acids may play different roles in the development of these diseases. However, the mechanisms linking individual saturated fatty acids to chronic diseases are largely unknown.

Adipose tissue, considered an endocrine organ by some scientists, not only stores fatty acids but also secretes adipokines, such as leptin, adiponectin, plasminogen activator inhibitor-1 (PAI-1), resistin, and visfatin [4]. Adipokines are involved in glucose metabolism (e.g., adiponectin, leptin, resistin, visfatin, and PAI-1 [5, 6]), inflammation (e.g., resistin and leptin [7]), reducing inflammation (e.g., adiponectin [5, 7]), coagulation (e.g., PAI-1 [6]), endothelial dysfunction (e.g., PAI-1 [8]), and feeding behavior (e.g., leptin [6]). In addition, adiponectin augments energy expenditure [9]. With regard to epidemiological evidence, high concentrations of leptin [10], resistin [11], visfatin [12], and PAI-1 [8, 13] have been associated with an increased risk of type 2 diabetes, whereas high adiponectin concentrations have been associated with a decreased risk of obesity [6] and type 2 diabetes [14, 15]. Furthermore, positive associations between leptin [16–18], PAI-1 [8] and cardiovascular disease have been documented. However, the overall impact of circulating fatty acid content on the effect of these adipokines remains unclear.

To our knowledge, only three studies have examined the association of circulating saturated fatty acids with adipokines, all of which studied European populations. One study showed that circulating odd-chain saturated fatty acids (15:0 and 17:0) were inversely associated with leptin and PAI-1 concentrations [19], whereas the second showed that circulating even-chain saturated fatty acids (14:0, 16:0, and 18:0) were not associated with leptin or adiponectin concentrations [20]. The third study showed that circulating palmitic acid (16:0) was inversely associated with adiponectin concentrations [21]. To date, no study has reported the association of circulating levels of saturated fatty acids with concentrations of resistin and visfatin. Importantly, Asian populations have relatively lower body mass compared with Western populations [22], and the effects of circulating saturated fatty acids on adipokines in Asian populations may differ from those in Western populations.

Here, we conducted a cross-sectional study of the associations of individual circulating saturated fatty acids in the serum phospholipid fraction with leptin, adiponectin, PAI-1, resistin, and visfatin in relatively healthy Japanese workers. Furthermore, we determined groupings of saturated fatty acids as additional exposures based on their potential biological actions. For example, odd-chain saturated fatty acids, including pentadecanoic acid (15:0) and heptadecanoic acid (17:0), reflect dietary consumptions (e.g. dairy fats [23–25]), whereas even-chain saturated fatty acids, including myristic acid (14:0), palmitic acid (16:0), and stearic acid (18:0), represent both *de novo* lipogenesis and dietary intake [26]. We hypothesized that high concentrations of even-chain saturated fatty acids would be associated with higher concentrations of leptin, PAI-1, resistin, and visfatin as well as lower adiponectin concentrations, and that odd-chain saturated fatty acids would be associated with lower concentrations.

## Materials and methods

### Study procedure and subjects

The study participants were employees of two municipal offices which were subject to health surveys, in July 2009 in one office and in November 2009 in the second. Details of the study procedure have been described elsewhere [27–29]. In brief, all full-time workers (n = 605) except those on prolonged sick leave or maternity leave were invited to participate in a survey of periodic health examinations. Among eligible employees, 567 participants (325 men and 242 women) aged 20–68 years participated in the survey (response rate 94%). Participants were asked to fill out the questionnaires before the checkup, and responses were checked by the research staff. We excluded 41 subjects with a history of cardiovascular disease (n = 11), cancer (n = 13), diabetes (n = 8), nephritis (n = 1), or chronic hepatitis (n = 3) or pregnancy (n = 8). Some participants met more than one exclusion criterion. Furthermore, we excluded those who had missing data regarding their serum fatty acid composition (n = 19) and those blood samples were collected in non-fasting conditions (n = 23). In total, 484 subjects (284 men and 200 women) were selected. Of these, we retained 482 subjects for the analysis of visfatin, after excluding those with visfatin levels above the upper detection limits (n = 2). The protocol of the study was approved by the ethics committee of the National Center for Global Health and Medicine, and written informed consent was obtained from each participant.

### Serum sampling

Participants were instructed to receive a checkup after an overnight fast. Venous blood (7 mL) was drawn into a vacuum tube and then conveyed to the laboratory in a cooler box. The blood was centrifuged at 4°C for 10 min at 1371×g, and the separated serum was divided into a maximum of six tubes (0.5 mL each). Five of these tubes were stored at −80°C (four tubes) or at −20°C (one tube, used specifically for the measurement of the fatty acid composition) until analysis.

### Measurement of fatty acid composition

Measurement of the fatty acid composition have been described in detail elsewhere [28, 29]. After serum lipids were extracted by the Folch method [30], phospholipids and other lipids were separated by thin-layer chromatography on silica gel G. The plates containing the serum lipid extracts were developed with petroleum ether/diethylether/acetic acid (82:18:1, vol/vol/vol). The fatty acids liberated from phospholipids were methylated with sulfuric acid/methanol (1:115, vol/vol), and the resulting fatty acid methyl esters were analyzed by gas chromatography (Shimadzu GC-17A; Shimadzu Corp., Kyoto, Japan). Fatty acid methyl esters were also analyzed using an Omegawax 320 Fused Silica Capillary Column (30 m long, 0.32 mm i.d., 0.25-mm film thickness) obtained from Supelco (Bellefonte, PA, USA). The fatty acid methyl ester values were calculated as the weight percentage based on each peak area. We identified 15 different fatty acids in phospholipids. These included five saturated fatty acids with relative concentrations higher than 0.05%, namely myristic acid (14:0), pentadecanoic acid (15:0), palmitic acid (16:0), heptadecanoic acid (17:0), and stearic acid (18:0). The intra-assay coefficient of variation values for the major fatty acid methyl esters were as follows: 14:0 (9.7%), 15:0 (8.7%), 16:0 (4.0%), 17:0 (3.4%), and 18:0 (1.6%) for phospholipids.

### Measurement of adipokines

To quantify the serum concentrations (pg/mL) of adiponectin, leptin, resistin, visfatin, and PAI-1, a Luminex suspension bead-based multiplexed array was performed using a Bio-Plex 3D suspension array system and Bio-Plex Pro Human Diabetes Assay Panel (Bio-Rad Laboratories, Hercules, CA). Intra-assay coefficient of variations were 12% for adiponectin, 11% for leptin, 8% for resistin, 19% for visfatin, and 21% for PAI-1 [27]. The reliability of multiplexed bead-based assays has been well demonstrated [31], although the measured values were not always identical to those generated by conventional ELISA assays. One study comparing several commercially available multiplex platforms concluded the Bio-Plex system was the most suitable for biomarker verification and validation [32].

### Other variables

The types of occupational and non-occupational physical activity (leisure-time and commuting from home to work) were surveyed in the questionnaire. Occupational physical activity was classified as sedentary work and active work. Non-occupational physical activity was recorded as the daily minutes spent walking or cycling during the respondent’s commute and the weekly hours engaged in each of five different activities in leisure (walking, low-, moderate-, and high-intensity activities, and gardening). Non-occupational physical activities were expressed as metabolic equivalent values and expressed as the sum of metabolic equivalents (MET) multiplied by the time (in hours) spent performing each activity. Alcohol consumption and smoking status were measured as the mean ethanol intake (grams per day) and the number of cigarettes smoked per day, respectively. Body height was measured to the nearest 0.1 cm, with the subject standing without shoes. Body weight in light clothes was measured to the nearest 0.1 kg. Body mass index (BMI) was calculated by dividing the weight by the height squared (kg/m^2^).

### Statistical analysis

The characteristics of the participants for each tertile of circulating saturated fatty acid were expressed as means (with standard deviation) for continuous variables and as percentages for categorical variables. Multiple linear regression analysis was performed to estimate the geometric means and 95% confidence intervals (CI) of the adipokine concentrations for each tertile of circulating saturated fatty acids. Before the analysis was performed, the adipokine concentrations were log-transformed to approximate normality. Model 1 adjusted for sex, age (years, continuous), and workplace (site A or B), and Model 2 additionally adjusted for BMI (kg/m^2^, continuous), occupational physical activity (sedentary work or active work), non-occupational physical activity (0, >0 to <5 MET-hr/week, 5 to <10 MET-hr/week, or ≥10 MET-hr/week), smoking status (never smokers, ex-smokers, current smokers consuming 1–19 cigarettes/day, or current smokers consuming ≥20 cigarettes/day), and alcohol consumption (no, >0 to <20, or ≥20 g ethanol/day). In the linear regression analysis, the trend associations were assessed by assigning the ordinal numbers 0–2 to the 3 categories of each fatty acid concentration. We made a post-hoc decision to create additional exposures based on the number of carbons of saturated fatty acids as follows: sum of the even-chain saturated fatty acids 14:0, 16:0, and 18:0; sum of the odd-chain saturated fatty acids 15:0 and 17:0. We repeated an analysis with adjustment for waist circumference instead of BMI (n = 300). Furthermore, we conducted sensitivity analyses by treating fatty acid data in mol% and by excluding individuals with data suggestive of inflammation (CRP level ≥0.3 mg/dL; n = 23) and those with a history of dyslipidemia (n = 9). We calculated Pearson’s correlation coefficients between concentrations of fatty acids and adipokines with adjustment for sex, age, workplace, BMI, occupational physical activity, non-occupational physical activity, smoking status, and alcohol consumption. We also examined the associations between concentrations of 15:0 and 17:0 and food intake, using Pearson correlation coefficients adjusted for age, sex, workplace, and total energy intake. Two-sided p-values <0.05 were regarded as statistically significant. Post hoc analysis reveals that the present data have a 77% power to detect a significant difference (effect size = 0.30) between the highest and lowest tertiles. All analyses were performed using the SAS statistical software package version 9.3 (SAS Institute Inc., Cary, NC, USA).

## Results

The mean age of the participants was 44.6 years for men and 43.1 years for women. The mean (standard deviation) concentrations of fatty acids was 0.25% (0.09%) for myristic acid (14:0), 0.17% (0.06%) for pentadecanoic acid (15:0), 30.74% (2.76%) for palmitic acid (16:0), 0.37% (0.07%) for heptadecanoic acid (17:0), and 15.05% (1.10%) for stearic acid (18:0). Participants with high odd-chain saturated fatty acid (15:0 and 17:0) concentrations were more likely to be young and female, but less likely to be a current smoker, current alcohol drinker or engaged in sedentary work or non-occupational physical activity compared with those with low concentrations of odd-chain saturated fatty acids (**Table 1**). Those with higher concentrations of odd-chain saturated fatty acids had lower BMIs than those with lower concentrations of odd-chain saturated fatty acids. By contrast, participants with high even-chain saturated fatty acids (14:0, 16:0, and 18:0) concentrations had higher BMIs than those with low concentrations of even-chain saturated fatty acids. Those with high concentrations of even-chain saturated fatty acids were more likely to be old, male, and a current smoker.

**Table 1:** Characteristics of participants^a^.

	15:0 + 17:0			14:0 +16:0 + 18:0		
	Tertile 1 (low)	Tertile 2	Tertile 3 (high)	Tertile 1 (low)	Tertile 2	Tertile 3 (high)
Number of subjects, n	162	160	162	161	162	161
Age (year)	44.8±11.2	43.7±10.5	43.6±10.5	42.5±10.3	42.8±11.0	46.7±10.4
Sex (% men)	75.3	64.4	36.4	42.9	67.9	65.2
Workplace (% site A) ^b^	29.6	25.6	29.0	0.0	35.8	48.5
Occupational physical activity (% sedentary work)	85.8	78.8	77.2	88.2	80.3	78.3
Non- occupational physical activity (% ≥5 metabolic equivalents-hr/wk)	38.3	37.5	28.4	34.8	33.3	36.0
Current smoking (%)	38.3	24.4	14.2	19.9	27.8	29.2
Current alcohol drinking (%)	77.2	57.5	49.4	57.1	66.1	60.9
Body mass index (kg/m^2^)	23.1±3.7	22.3±2.9	21.8±3.0	21.5±2.8	22.4±3.2	23.3±3.5

^a^Mean values and standard deviation for continuous variables and number of participants (proportion) for categorical variables.

^b^Survey conducted in July 2009.

**Fig 1:**
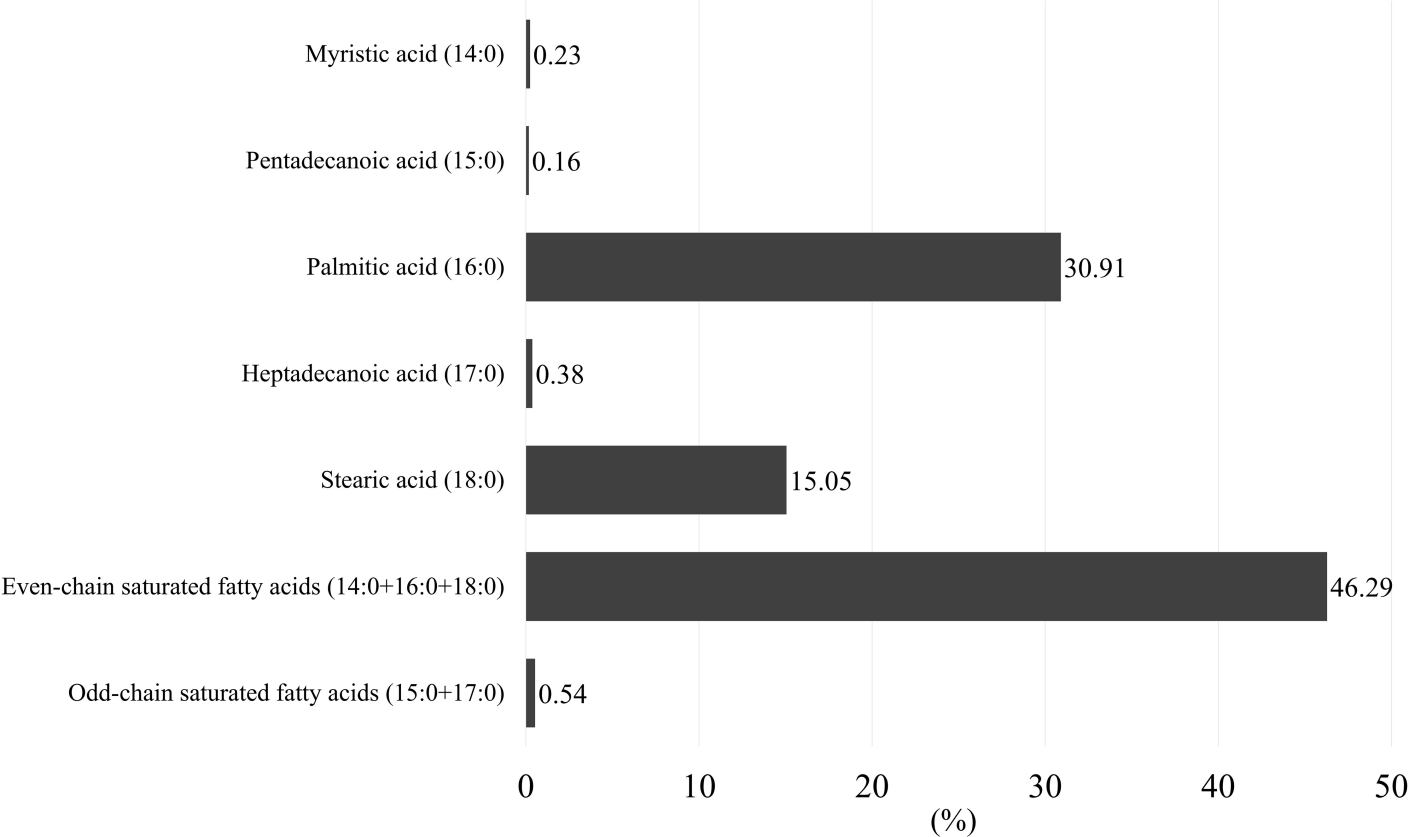
Median proportions of saturated fatty acids (%).

The proportions of each saturated fatty acid were as shown in **Fig 1**. Majority of saturated fatty acids was consisted of even-chain saturated fatty acids (46.3%), especially palmitic acid (16:0) (30.9%).

The relationship between odd-chain saturated fatty acids in serum phospholipids and adipokines is shown in **Table 2**. In our model adjusted for age, sex, workplace, physical activity, smoking status, alcohol consumption, and BMI, the sum of odd-chain saturated fatty acids (15:0+17:0) showed decrements of leptin concentration from 1.91 ng/mL (95% CI: 1.71–2.13) in those with the lowest tertile to 1.62 ng/mL (95% CI: 1.45–1.80) in those with the highest tertile (P for trend = 0.048). Similarly, the PAI-1 concentrations decreased from 32.6 ng/mL (95% CI: 31.1–34.2) and 31.6 ng/mL (95% CI: 30.1–33.2) in those with the lowest tertile to 29.7 ng/mL (95% CI: 28.3–31.1) and 29.1 ng/mL (95% CI: 27.8–30.6) in those with the highest tertile of pentadecanoic acid (15:0) and the highest sum of the odd-chain saturated fatty acids (15:0+17:0), respectively (P for trend = 0.007 and 0.02, respectively). In addition, high concentrations of pentadecanoic acid (15:0) were associated with lower adiponectin concentrations (P for trend = 0.01). However, visfatin was positively associated with pentadecanoic acid (15:0) and the sum of odd-chain saturated fatty acids (15:0+17:0) (P for trend = 0.001 and 0.04, respectively).

**Table 2:** Multivariate adjusted geometric means (and 95% confidence interval) for adipokines among phospholipids saturated fatty acids.

	Fatty acid (%), median	Multivariate adjusted geometric means (95% confidence interval) ^a^				
		Leptin (ng/ml)	Adiponectin (μg/ml)	PAI-1 (ng/ml)	Resistin (ng/ml)	Visfatin (ng/ml)
***Myristic acid (14*:*0)***						
Tertile 1 (low)	0.18	1.53 (1.37–1.70)	5.34 (4.83–5.91)	30.4 (29.1–31.9)	3.15 (2.90–3.42)	0.82 (0.72–0.94)
Tertile 2	0.23	1.87 (1.69–2.08)	4.80 (4.35–5.30)	30.4 (29.0–31.8)	3.17 (2.92–3.44)	1.01 (0.88–1.14)
Tertile 3 (high)	0.33	1.84 (1.65–2.05)	4.37 (3.94–4.83)	30.9 (29.5–32.4)	3.25 (2.99–3.53)	1.08 (0.95–1.23)
P trend		**0.02**	**0.007**	0.66	0.61	**0.01**
***Pentadecanoic acid (15*:*0)***						
Tertile 1 (low)	0.13	1.83 (1.64–2.04)	5.28 (4.77–5.85)	32.6 (31.1–34.2)	3.16 (2.91–3.44)	0.83 (0.72–0.94)
Tertile 2	0.16	1.73 (1.55–1.92)	4.91 (4.44–5.43)	29.5 (28.2–30.9)	3.27 (3.01–3.56)	0.94 (0.83–1.07)
Tertile 3 (high)	0.21	1.66 (1.49–1.85)	4.33 (3.91–4.79)	29.7 (28.3–31.1)	3.13 (2.88–3.41)	1.15 (1.00–1.31)
P trend		0.23	**0.01**	**0.007**	0.88	**0.001**
***Palmitic acid (16*:*0)***						
Tertile 1 (low)	27.96	1.85 (1.65–2.08)	5.28 (4.74–5.87)	31.2 (29.6–32.7)	3.02 (2.76–3.30)	0.81 (0.70–0.93)
Tertile 2	30.91	1.65 (1.49–1.84)	5.21 (4.72–5.75)	30.7 (29.3–32.1)	3.16 (2.91–3.43)	0.99 (0.87–1.13)
Tertile 3 (high)	33.56	1.71 (1.53–1.92)	4.07 (3.67–4.52)	29.9 (28.5–31.4)	3.40 (3.12–3.71)	1.11 (0.97–1.27)
P trend		0.39	**0.001**	0.26	0.07	**0.003**
						(continued)
***Heptadecanoic acid (17*:*0)***						
Tertile 1 (low)	0.30	1.93 (1.72–2.15)	4.56 (4.10–5.06)	30.9 (29.4–32.4)	3.06 (2.81–3.34)	0.94 (0.82–1.08)
Tertile 2	0.38	1.64 (1.48–1.82)	5.04 (4.57–5.57)	31.5 (30.1–33.0)	3.55 (3.27–3.84)	0.96 (0.84–1.09)
Tertile 3 (high)	0.43	1.66 (1.49–1.85)	4.87 (4.39–5.40)	29.3 (28.0–30.7)	2.98 (2.74–3.24)	0.99 (0.87–1.14)
P trend		0.08	0.41	0.13	0.57	0.56
***Stearic acid (18*:*0)***						
Tertile 1 (low)	14.01	1.58 (1.42–1.76)	4.82 (4.36–5.33)	31.0 (29.6–32.5)	3.17 (2.92–3.45)	0.97 (0.85–1.10)
Tertile 2	15.05	1.80 (1.62–1.99)	4.91 (4.45–5.42)	29.9 (28.6–31.3)	3.22 (2.96–3.49)	0.91 (0.80–1.03)
Tertile 3 (high)	16.05	1.85 (1.66–2.06)	4.73 (4.27–5.23)	30.8 (29.4–32.3)	3.18 (2.92–3.45)	1.01 (0.89–1.15)
P trend		**0.04**	0.79	0.85	0.98	0.67
***Even-chain saturated fatty acids (14*:*0+16*:*0+18*:*0)***						
Tertile 1 (low)	43.11	1.81 (1.61–2.03)	5.35 (4.80–5.97)	30.2 (28.7–31.8)	2.96 (2.70–3.24)	0.78 (0.68–0.90)
Tertile 2	46.29	1.70 (1.53–1.89)	4.97 (4.50–5.50)	31.7 (30.3–33.2)	3.23 (2.98–3.51)	1.03 (0.90–1.17)
Tertile 3 (high)	48.69	1.71 (1.53–1.92)	4.21 (3.79–4.68)	29.9 (28.4–31.3)	3.39 (3.11–3.70)	1.11 (0.97–1.28)
P trend		0.57	**0.003**	0.66	**0.048**	**0.001**
***Odd-chain saturated fatty acids (15*:*0+17*:*0)***						
Tertile 1 (low)	0.45	1.91 (1.71–2.13)	4.74 (4.26–5.26)	31.6 (30.1–33.2)	3.03 (2.78–3.30)	0.84 (0.73–0.97)
Tertile 2	0.54	1.70 (1.53–1.89)	4.91 (4.44–5.43)	31.0 (29.7–32.5)	3.55 (3.28–3.86)	1.01 (0.89–1.16)
Tertile 3 (high)	0.63	1.62 (1.45–1.80)	4.82 (4.34–5.35)	29.1 (27.8–30.6)	3.02 (2.77–3.28)	1.04 (0.91–1.19)
P trend		**0.048**	0.84	**0.02**	0.91	**0.04**

PAI-1: plasminogen activator inhivitor-1

^a^Adjusted for sex, age (years, continuous), workplace (A or B), sedentary work (yes or no), non-occupational physical activity (0, >0 to <5, or ≥5 metabolic equivalents-hr/wk), smoking status (never, past, current smoking for 1–19 cigarettes or ≥20 cigarettes), current alcohol consumption (no, <20, or ≥20 g ethanol/day), and body mass index (kg/m^2^, continuous).

The relationships between even-chain saturated fatty acids in serum phospholipids and adipokines are also shown in **Table 2**. After adjustment for age, sex, workplace, physical activity, smoking status, alcohol consumption, and BMI, leptin concentrations increased from 1.53 ng/mL (95% CI: 1.37–1.70) in those with the lowest tertile of myristic acid (14:0) to 1.84 ng/mL (95% CI: 1.65–2.05) in those with the highest tertile (P for trend = 0.02). Similarly, the leptin concentrations increased from 1.58 ng/mL (95% CI: 1.42–1.76) in those with the lowest tertile of stearic acid (18:0) to 1.85 ng/mL (95% CI: 1.66–2.06) in those with the highest (P for trend = 0.04). Resistin concentrations were marginally significantly positively associated with palmitic acid (16:0) concentrations (P for trend = 0.07), and increased from 2.96 ng/mL (95% CI: 2.70–3.24) in those with the lowest tertile of the sum of even-chain saturated fatty acids (14:0+16:0+18:0) to 3.39 ng/ml (95% CI: 3.11–3.70) in those with the highest tertile (P for trend = 0.048). High concentrations of myristic (14:0) and palmitic (16:0) acids and the sum of even-chain saturated fatty acids (14:0+16:0+18:0) were associated with higher concentrations of visfatin (P for trend = 0.01, 0.003, and 0.001, respectively). In contrast, adiponectin concentrations decreased from 5.34 μg/mL (95% CI: 4.83–5.91) and 5.28 μg/mL (95% CI: 4.74–5.87) in those with the lowest tertile to 4.37 μg/mL (95% CI: 3.94–4.83) and 4.07 μg/mL (95% CI: 3.67–4.52) in those with the highest tertile of myristic acid (14:0) and palmitic acid (16:0), respectively (P for trend = 0.007 and 0.001, respectively). The sum of even-chain saturated fatty acids (14:0+16:0+18:0) was also inversely associated with the adiponectin concentrations (P for trend = 0.003). However, PAI-1 was not associated with any even-chain saturated fatty acids.

Similar associations were observed when fatty acid data were expressed in mol% (**S1 Table**). The associations did not materially change after adjusting for waist circumference (data not shown) or excluding individuals with a CRP level ≥0.3 mg/dL (data not shown) or those with a history of dyslipidemia (data not shown). Moreover, similar associations were also seen in an analysis which treated fatty acids as a continuous variable (data not shown).

Some foods showed weak or moderate correlations with pentadecanoic acid (15:0): Western confectionaries (r = 0.12), Japanese confectionaries (r = 0.10), and alcoholic beverages (r = −0.19). Similarly, foods correlating with heptadecanoic acid (17:0) were eggs (r = 0.17), soft drinks (r = 0.15), fish (r = 0.14), mayonnaise (r = 0.10), milk and dairy products (r = 0.09), noodles (r = −0.14), and alcoholic beverages (r = −0.28).

## Discussion

To date, a recent meta-analysis from prospective cohort studies showed that overall saturated fats intake is not associated with risk of cardiovascular disease, or type 2 diabetes [33]. In this study, we found that circulating even- and odd-chain saturated fatty acids were differentially associated with adipokine profile, which has been shown to be related to the risk of cardiovascular disease or type 2 diabetes [8, 10, 13, 16–18]. In this study of a cohort of Japanese workers, circulating odd-chain saturated fatty acids, including pentadecanoic acid (15:0) and heptadecanoic acid (17:0), were inversely associated with the concentrations of leptin, PAI-1, and adiponectin, whereas odd-chain saturated fatty acids were positively associated with visfatin. Circulating even-chain saturated fatty acids, including myristic acid (14:0), palmitic acid (16:0), and stearic acid (18:0), were positively associated with the concentrations of leptin, resistin, and visfatin, but were inversely associated with adiponectin. We therefore confirmed the applicability of the evidence from Western studies to Japanese individuals. To our knowledge, this is the first study to investigate adipokine concentrations in relation to circulating even- and odd-chain saturated fatty acids in a single study.

The observed associations of odd-chain saturated fatty acids (15:0+17:0) with leptin and PAI-1 concentrations agree with those of a cross-sectional analysis of baseline data of a prospective case-control study in Sweden (78 acute myocardial infarction cases and 156 controls) [19], indicating that the relationships between odd-chain saturated fatty acids and these adipokines are consistent irrespective of circulating levels of odd-chain saturated fatty acids (0.54% in the present study vs 0.67% in the Swedish study). However, that study did not consider potential confounding variables, such as physical activity, smoking status, or alcohol consumption. Our present findings are consistent with our findings that a Westernized breakfast pattern, which was characterized by higher intake of confectionaries, bread, and milk and yogurt but lower intake of alcoholic beverages and rice, was inversely associated with leptin and PAI-1 concentrations [34]. Foods characterizing the Westernized breakfast pattern in that study were also correlated with odd-chain saturated fatty acids in the present study (e.g., positive correlation with confectionaries and milk and dairy products but inverse correlation with alcoholic beverages). Our findings are also compatible with those linking odd-chain saturated fatty acids to cardiovascular disease [2, 19] and diabetes [3, 35, 36], the risks of which have been associated with leptin [10, 16–18] and PAI-1 [8, 13]. Specifically, Warensjö et al. found a decreased risk of myocardial infarction among those who had high plasma levels of phospholipids, pentadecanoic acid (15:0), heptadecanoic acid (17:0), and the sum of these fatty acids [19]. In the EPIC and the Norfolk Prospective Study, odd-chain saturated fatty acids concentrations were associated with a decreased risk of coronary heart disease [2]. The EPIC-InterAct case-cohort study showed that these fatty acids were inversely associated with diabetes risk [3], and two other prospective studies also found that a decreased risk of incident diabetes was associated with higher proportions of pentadecanoic acid (15:0) [35, 36] and heptadecanoic acid (17:0) [35]. Our results, together with existing data in Western populations, suggest that odd-chain saturated fatty acids are associated with lower levels of leptin and PAI-1, which may partly account for the lower risk of metabolic and cardiovascular diseases in individuals with higher concentrations of odd-chain saturated fatty acids.

We found a positive association of even-chain saturated fatty acids with leptin, resistin, and visfatin concentrations and an inverse association with adiponectin concentrations. The present inverse association with adiponectin is consistent with that of a cross-sectional study in a Spanish population [21], whereas our findings are at odds with the findings of a cross-sectional study by Santos et al., who reported no association between the sum of even-chain saturated fatty acids (12:0+14:0+16:0+18:0) and leptin or adiponectin concentrations among inhabitants in Portugal [20]. With regard to resistin and visfatin, to our knowledge, no study has examined their association with even-chain saturated fatty acids. However, our findings may be supported by the results of large studies of type 2 diabetes risk [3, 37] and coronary heart disease [2], which have been associated with higher concentrations of leptin [10], resistin [11], and visfatin [12] and with lower concentrations of adiponectin [14]. Likewise, the EPIC-InterAct case-cohort study reported a positive association between plasma phospholipids, even-chain saturated fatty acids, and incident diabetes [3]. In addition, the Cardiovascular Health Study, which is a community-based cohort of older adults in the U.S., showed that circulating palmitic acid (16:0) and stearic acid (18:0) were positively associated with diabetes risk, adiposity, inflammation, and insulin resistance [37]. Furthermore, the EPIC and the Norfolk Prospective Study showed that even-chain saturated fatty acids concentrations were associated with an increased risk of coronary heart disease [2]. The available epidemiological data suggest that higher concentrations of even-chain saturated fatty acids are associated with an unfavorable adipokine profile, which may play a role in the development of metabolic disorders.

The biological mechanisms underlying the associations between individual saturated fatty acids and adipokines are unclear, but some pathways have been suggested. An odd-chain saturated fatty acid has a lower melting point, which is a determinant of the alteration of fluidity, than its next lower even-numbered homolog [38, 39], and therefore pentadecanoic acid (15:0) may increase the fluidity of acyl chains. Alteration of membrane fluidity such as in the hypothalamus may enhance leptin delivery (mediated by OB-R) [40] or leptin receptor activity, and increased leptin sensitivity in the central nervous system may lead to a decrease in plasma leptin concentration. Another pathway may exist. Interestingly, valine and isoleucine catabolism contributes significantly to lipogenic propionyl-CoA pool, which may result in high rates of odd-chain fatty acid synthesis in 3T3-L1 adipocytes [41]. It has been reported that branched chain amino acids (BCAA) may be implicated in obesity, insulin resistance [42], and type 2 diabetes [43], and BCCA catabolism also accelerates adipocyte differentiation [44]. Although we did not measure BCAAs in our panel, it may be interesting to speculate that the changes in fatty acid composition which we observed here may have biological implications in adipose tissues in relation to BCAA catabolism. With regard to even-chain saturated fatty acids, in C/EBPα null mice, palmitic acid (16:0) decreased the expression of adiponectin mRNA *via* phosphorylation of peroxisome proliferator-activated receptor-γ (PPAR-γ) on Ser273, which may stimulate lysosomal degradation of newly synthesized adiponectin [45]. Additionally, even-chain saturated fatty acids, including lauric acid (12:0) and palmitic acid (16:0), induce macrophage inflammation, such as that involving nuclear factor-κB, which appears to be mediated, in part, by Toll-like receptor 4 signaling [46, 47]. This macrophage inflammation decreases PPAR-γ activity. Given that PPAR-γ inhibits leptin gene expression in cell-culture experiments [48], the decrease in PPAR-γ activity might lead to increased leptin concentrations.

The major strengths of this study include its high participation rate (94%) in a well-defined working population, use of relatively stable biomarkers of fatty acid status and adipokines, and adjustment for potentially important confounders. The study also has several limitations. First, we cannot infer causality due to the cross-sectional nature of the study design. Second, we measured the fatty acid composition and adipokines at a single time point, which might not represent long-term status. However, a single time point measurement of adipokine concentrations is known to be highly correlated with the mean of the remaining three seasonal samples [49]. Third, we measured fatty acids in serum phospholipids, which does not reflect long-term dietary intake like those in erythrocytes [50]. A greater probability of misclassification in short-term assessment of exposure might distort the associations between fatty acid composition and adipokines toward the null (underestimation of the true magnitude of the association). Fourth, because circulating levels of even-chain saturated fatty acids reflect both dietary intake and *de novo* lipogenesis [26], the observed associations between circulating even-chain saturated fatty acids and adipokines cannot be directly linked to dietary recommendations for saturated fatty acid intake. Fifth, we made a number of comparisons, and some associations with statistical significance may be due to chance. For example, contrary to our hypothesis, we observed increased concentrations of visfatin and decreased concentrations of adiponectin among those with high concentrations of odd-chain saturated fatty acids. Sixth, although we adjusted for potentially important confounding variables, the possibility of residual confounding cannot be excluded. For example, we adjusted for BMI as an indicator of adiposity. However, BMI does not differentiate between lean mass and fat mass and provides no data for visceral adiposity, which is more closely associated with metabolic complications than overall adiposity [51], thus leaving open the possibility of residual confounding after BMI adjustment. Finally, our study cohort consisted of apparently healthy Japanese workers. The present findings might not be applicable to populations with a different background.

In conclusion, this study suggests that odd-chain saturated fatty acids are associated with a favorable serum adipokine profile, whereas even-chain saturated fatty acids are associated with an unfavorable profile. Prospective studies are required to confirm the associations between adipokines and individual saturated fatty acids in this cross-sectional study.

## Supporting information

S1 Table Multivariate adjusted geometric means (and 95% confidence interval) for adipokines among phospholipids saturated fatty acids (mol%). (DOCX)
