# Supplementary material for: Even- and odd-chain saturated fatty acids in serum phospholipids are differentially associated with adipokines
Source: PLoS One. 2017 May 26;12(5):e0178192. doi: 10.1371/journal.pone.0178192 (PMC5446160; doi:10.1371/journal.pone.0178192)
Supplement: S1 Table — (DOCX) [file pone.0178192.s001.docx]

S1 Table Multivariate adjusted geometric means (and 95% confidence interval) for adipokines among phospholipids saturated fatty acids (mol%)

|  | Fatty acid (mol%) | Multivariate adjusted geometric means (95% confidence interval) * | | | | |
| --- | --- | --- | --- | --- | --- | --- |
|  |  | Leptin  (ng/ml) | Adiponectin  (µg/ml) | PAI-1  (ng/ml) | Resistin  (ng/ml) | Visfatin  (ng/ml) |
| ***Myristic acid (14:0)*** |  |  |  |  |  |  |
| Tertile 1 (low) | 0.21 | 1.54 (1.38-1.71) | 5.36 (4.85-5.94) | 30.61 (29.21-32.08) | 3.13 (2.88-3.41) | 0.80 (0.70-0.91) |
| Tertile 2 | 0.28 | 1.84 (1.66-2.04) | 4.85 (4.40-5.35) | 30.18 (28.83-31.58) | 3.19 (2.94-3.46) | 1.03 (0.91-1.17) |
| Tertile 3 (high) | 0.39 | 1.85 (1.66-2.06) | 4.31 (3.89-4.76) | 30.94 (29.53-32.42) | 3.25 (2.99-3.53) | 1.09 (0.95-1.24) |
| P trend |  | 0.02 | 0.003 | 0.76 | 0.56 | 0.001 |
| ***Pentadecanoic acid (15:0)*** |  |  |  |  |  |  |
| Tertile 1 (low) | 0.14 | 1.82 (1.64-2.04) | 5.27 (4.76-5.84) | 32.34 (30.86-33.90) | 3.17 (2.91-3.45) | 0.83 (0.73-0.95) |
| Tertile 2 | 0.19 | 1.75 (1.58-1.95) | 4.89 (4.43-5.40) | 29.98 (28.65-31.36) | 3.28 (3.02-3.56) | 0.95 (0.83-1.08) |
| Tertile 3 (high) | 0.24 | 1.64 (1.47-1.83) | 4.34 (3.92-4.81) | 29.48 (28.13-30.90) | 3.12 (2.87-3.40) | 1.13 (0.99-1.30) |
| P trend |  | 0.19 | 0.01 | 0.01 | 0.83 | 0.002 |
|  |  |  |  |  |  |  |
|  |  |  |  |  |  |  |
| ***Palmitic acid (16:0)*** |  |  |  |  |  |  |
| Tertile 1 (low) | 30.37 | 1.86 (1.65-2.08) | 5.30 (4.76-5.90) | 31.28 (29.76-32.88) | 3.00 (2.75-3.28) | 0.80 (0.70-0.92) |
| Tertile 2 | 33.44 | 1.66 (1.49-1.84) | 5.18 (4.70-5.72) | 30.84 (29.46-32.29) | 3.14 (2.89-3.40) | 0.99 (0.87-1.13) |
| Tertile 3 (high) | 36.21 | 1.71 (1.52-1.91) | 4.08 (3.68-4.53) | 29.62 (28.22-31.10) | 3.44 (3.16-3.75) | 1.12 (0.97-1.28) |
| P trend |  | 0.36 | 0.001 | 0.14 | 0.04 | 0.002 |
| ***Heptadecanoic acid (17:0)*** |  |  |  |  |  |  |
| Tertile 1 (low) | 0.31 | 1.94 (1.73-2.16) | 4.59 (4.13-5.10) | 30.93 (29.48-32.45) | 3.10 (2.85-3.38) | 0.97 (0.84-1.11) |
| Tertile 2 | 0.39 | 1.62 (1.46-1.80) | 5.00 (4.53-5.53) | 31.44 (30.04-32.92) | 3.48 (3.21-3.78) | 0.91 (0.80-1.04) |
| Tertile 3 (high) | 0.44 | 1.67 (1.50-1.86) | 4.88 (4.40-5.40) | 29.38 (28.04-30.79) | 3.00 (2.76-3.26) | 1.01 (0.89-1.16) |
| P trend |  | 0.09 | 0.45 | 0.13 | 0.51 | 0.60 |
| ***Stearic acid (18:0)*** |  |  |  |  |  |  |
| Tertile 1 (low) | 13.75 | 1.57 (1.41-1.74) | 4.74 (4.29-5.24) | 30.79 (29.40-32.23) | 3.18 (2.93-3.45) | 0.99 (0.87-1.13) |
| Tertile 2 | 14.75 | 1.80 (1.62-2.00) | 4.88 (4.42-5.39) | 30.00 (28.67-31.39) | 3.17 (2.93-3.44) | 0.91 (0.80-1.04) |
| Tertile 3 (high) | 15.78 | 1.86 (1.67-2.07) | 4.84 (4.38-5.36) | 30.95 (29.56-32.42) | 3.21 (2.96-3.49) | 0.99 (0.87-1.13) |
| P trend |  | 0.03 | 0.77 | 0.88 | 0.87 | 1.00 |
|  |  |  |  |  |  |  |
|  |  |  |  |  |  |  |
| ***Even-chain saturated fatty acids (14:0+16:0+18:0)*** |  |  |  |  |  |  |
| Tertile 1 (low) | 45.48 | 1.80 (1.60-2.02) | 5.35 (4.80-5.97) | 30.58 (29.07-32.17) | 2.97 (2.71-3.25) | 0.75 (0.65-0.87) |
| Tertile 2 | 48.58 | 1.69 (1.52-1.88) | 4.97 (4.50-5.49) | 31.60 (30.18-33.08) | 3.21 (2.96-3.48) | 1.04 (0.92-1.19) |
| Tertile 3 (high) | 51.05 | 1.73 (1.54-1.93) | 4.21 (3.79-4.68) | 29.58 (28.17-31.05) | 3.40 (3.12-3.71) | 1.13 (0.99-1.30) |
| P trend |  | 0.69 | 0.003 | 0.32 | 0.048 | 0.0003 |
| ***Odd-chain saturated fatty acids (15:0+17:0)*** |  |  |  |  |  |  |
| Tertile 1 (low) | 0.47 | 1.90 (1.70-2.13) | 4.75 (4.28-5.28) | 31.59 (30.10-33.16) | 3.01 (2.77-3.28) | 0.85 (0.74-0.97) |
| Tertile 2 | 0.57 | 1.68 (1.51-1.86) | 4.92 (4.45-5.44) | 30.82 (29.45-32.26) | 3.52 (3.25-3.82) | 1.01 (0.89-1.15) |
| Tertile 3 (high) | 0.68 | 1.64 (1.47-1.84) | 4.79 (4.31-5.32) | 29.35 (27.98-30.79) | 3.05 (2.80-3.32) | 1.04 (0.91-1.19) |
| P trend |  | 0.08 | 0.93 | 0.04 | 0.89 | 0.04 |

PAI-1: plasminogen activator inhivitor-1

*Adjusted for sex, age (years, continuous), workplace (A or B), sedentary work (yes or no), non-occupational physical activity (0, >0 to <5, or ≥5 metabolic equivalents-hr/wk), smoking status (never, past, current smoking for 1-19 cigarettes or ≥20 cigarettes), current alcohol consumption (no, <20, or ≥20 g ethanol/day), and body mass index (kg/m^2^, continuous).
